# Further Advantages of a Unique Author Identification Number

**DOI:** 10.1371/journal.pmed.0030368

**Published:** 2006-08-29

**Authors:** Etienne Joly

**Affiliations:** Institut de Pharmacologie et de Biologie Structurale, UMR CNRS 5089 Toulouse, France

I am in complete agreement with the suggestion by Matthew Falagas [1] that a unique author identification number (UAIN) would represent a major improvement for the use of databases of scientific publications. In this regard, I perceive that he has not mentioned several other important advantages that a UAIN system would provide, and that are worth pointing to:

1. When looking up someone's publications, the fact that the last name of a given person can vary from one paper to another can be as much of a problem as that of multiple authors with the same name. For example, these variations include women who change their last name after getting married (or divorced), middle initials that are sometimes included or omitted, translations from non-Roman alphabets that result in variable spellings, and people with last names composed of several terms that can sometimes appear in databases as split or truncated.

2. Contrarily to M. Falagas, I do not see any good reason why a UAIN system could not be retroactive. It is clearly in every scientist's interest to facilitate the job of other people who want to look up their work. I therefore believe that authors could be asked to register for a UAIN, and to validate their list of publications themselves, retroactively. Even for the most productive scientists, this would take only a few minutes, and the fact that they had registered for a UAIN allowing users to trace their whole list of publications could then be indicated in the display of search results from the various bibliographic databases. I also do not see any reason for “hiding” this UAIN. I suggest that it could be designed to be quite simple to remember and to communicate to others, for example: the first four or five letters of the last name followed by the initial of the first name followed by the year of first scientific publication followed by an incremental number depending on order of registration (my UAIN would be JOLY-E-89-01). It would therefore be something quite comparable in length and spirit to a car's licence plate and, like UK licence plates, it would provide an interesting clue regarding the seniority of its bearer.

3. This type of UAIN would therefore provide a very simple way to assess a person's productivity. It would also provide a very useful means to assess the actual impact of their work in terms of citations, by discriminating between self-citation and citations by others.

Today, most people are evaluated via the impact factor of the journals in which they have managed to publish their work, and not by the actual impact of the papers themselves. Although most scientists acknowledge that this is an extremely crude and unfair way of assessing the quality of someone's production, the impact factor lives on. By providing the simple means to track someone's bibliographic record and thus facilitate the evaluation of their productivity, I believe that the introduction of a UAIN system will not only help the scientific community to exploit bibliographic databases more efficiently, but also represent a major step towards getting rid of the despotic domination of the dreaded impact factors of journals as a means to evaluate the quality of scientific papers.
